# Vulcanizing Oven

**Published:** 1862-02

**Authors:** 


### VULCANIZING OVEN.

WE have for the last three months been using "Hayes' Vulcanizing Oven," and esteem it the best apparatus for the purpose we have ever seen: at least it has some advantages over all others. It is the smallest and most compact vulcanizer made. In use there is no escape of steam or vapor of any kind, it is neat in appearance, and may be used anywhere without inconvenience. It accomplishes the work in less time than any other, and is perfectly manageable. The thermometer is set and so guarded, that it is not liable to be broken; indeed, there is very little liability in any part of the apparatus to become impaired. It can be used with either gas or alcohol, and does not consume one-fourth part as much of either as any other machine.

We do not think any one who becomes accustomed to its use would for any consideration return to the awkward and clumsy affairs heretofore in use. T.

